# Associations of Dietary and Circulating Vitamin E Level With Metabolic Syndrome. A Meta-Analysis of Observational Studies

**DOI:** 10.3389/fnut.2021.783990

**Published:** 2021-12-08

**Authors:** Yi Zhang, Jun Ding, Hongbin Guo, Ze Liu, Qi Liu, Yusheng Li, Dianzhong Zhang, Jieyu Liang

**Affiliations:** ^1^Department of Orthopaedics, Xiangya Hospital, Central South University, Changsha, China; ^2^National Clinical Research Center for Geriatric Disorders, Xiangya Hospital, Central South University, Changsha, China; ^3^Changsha Social Work College, Changsha, China; ^4^Center for Teaching and Research of Advanced Mathematics, School of Mathematics and Statistics, Central South University, Changsha, China

**Keywords:** dietary vitamin E, circulating vitamin E, metabolic syndrome, meta-analysis, observational studies

## Abstract

**Objective:** The associations of dietary and circulating vitamin E level with metabolic syndrome (MetS) remains conflicting. This meta-analysis of observational study was therefore employed to investigate the issue above.

**Methods:** The PubMed, Web of Science and Embase database were searched up to April 2021. The observational studies on the associations of dietary and circulating vitamin E level with MetS were specified. The pooled relative risk (RR) of MetS for the highest vs. lowest dietary and circulating vitamin E level, and the standard mean difference (SMD) of dietary and circulating vitamin E level for MetS vs. control subjects, were calculated.

**Results:** A total of 25 observational studies with 51,276 participants, were included in this meta-analysis. The overall multi-variable adjusted RR demonstrated that the dietary vitamin E level was inversely associated with MetS (RR = 0.92, 95%CI: 0.85–1.00; *P* = 0.044). In addition, the dietary vitamin E level in MetS was also lower than that in control subjects according to the overall combined SMD (SMD = −0.08, 95%CI: −0.14 to −0.02; *P* = 0.024). On the other hand, the overall multi-variable adjusted RR showed no significant relationship between the circulating vitamin E level and MetS (RR = 1.46, 95%CI: 0.85–2.48; *P* = 0.17). However, the circulating vitamin E level in MetS was lower than that in control subjects according to the overall combined SMD (SMD = −0.58, 95%CI: −1.04 to −0.13; *P* = 0.013).

**Conclusions:** The results of this meta-analysis suggest that the dietary vitamin E level is inversely associated with MetS. On the other hand, current evidence is still insufficient to conclude a relationship between the circulating vitamin E level and MetS. More well-designed prospective cohort studies are needed to address the issues further.

## Introduction

Metabolic syndrome (MetS) is a pathological state characterized by the following clinical features: elevated waist circumference, blood pressure, fasting blood glucose, triglycerides and reduced high-density lipoprotein cholesterol (1). As a common risk factor for cardiovascular disease, diabetes and death, MetS has gradually become a major global public health issue (2). With the deepening of our knowledge, nutritional factors are considered to be involved in MetS (3–7).

As a fat-soluble vitamin with antioxidant properties, vitamin E is considered to be involved in signal transduction, gene expression and immunomodulatory capabilities (8). Higher vitamin E level was reported to be inversely associated with hypertension and diabetes (9–11). Moreover, a meta-analysis of randomized controlled trials demonstrated that vitamin E supplementation was beneficial for subclinical inflammation in adults (12). Experimental evidence also indicated that vitamin E supplementation could ameliorate hypertension and diabetes (13–15), and decrease the total serum cholesterol and lipid peroxides level (16) in animal model.

A number of observational studies have examined the associations of dietary and circulating vitamin E level with MetS. However, their results are still conflicting (17–41). The present meta-analysis of observational studies was therefore employed to address the issues. It was hypothesized that both dietary and circulating vitamin E was inversely associated with MetS.

## Materials and Methods

### Search Strategy

This meta-analysis was performed in accordance with the Preferred Reporting Items for Systematic Reviews and Meta-analyses (PRISMA) guidelines (42). The PubMed, Web of Science and Embase electronic database were searched during April 2021 by using a combination of keywords and in-text words related to metabolic syndrome (“metabolic syndrome”) and vitamin E (“vitamin E”, “tocopherol”) (43). No language restrictions were set in the search strategy. The titles and abstracts of all articles were screened firstly, and then the full articles were read to include the eligible studies. To identify the additional studies, the reference lists for the retrieved articles were also reviewed.

### Study Selection

The titles, abstracts and full texts of all retrieved studies were reviewed by two researchers (YZ and DZZ) independently. Disagreements were resolved by discussions. The included studies were required to meet the following criteria: (1) observational studies; (2) the associations of dietary and circulating vitamin E level with MetS were reported; (3) relative risk (RR), odds ratio (OR) or standard mean difference (SMD) with 95% confidence interval (CI) were reported. The exclusion criteria were listed as follows: (1) duplicated or irrelevant articles; (2) reviews, letters or case reports; (3) randomized controlled trials; and (4) non-human studies.

### Data Extraction

The data were extracted by two researchers (YZ and DZZ) independently, and disagreements were resolved by discussions. The information about first author, year of publication, location, age, gender, sample size, study design, adjustments, exposure, category of exposure, effect estimates and diagnostic criteria of MetS, was collected respectively. The corresponding effect estimates adjusted for the maximum number of confounding variables with 95% CIs for the highest vs. lowest dietary and circulating vitamin E level were extracted. Moreover, the dietary and circulating vitamin E level (mean ± SD) was also extracted to calculate the SMD (MetS vs. control).

### Quality Assessment

Quality assessment was conducted according to the Newcastle-Ottawa (NOS) criteria for non-randomized studies, which was based on three broad perspectives: the selection process of study cohorts, the comparability among different cohorts and the identification of exposure or outcome of study cohorts. Disagreements with respect to the methodological quality were resolved by discussion and mutual-consultation. A study awarded seven or more stars was considered as a high-quality study (44).

### Statistical Analyses

The RR for MetS and SMD for both dietary and circulating vitamin E level were the outcome measures in our study. The *I*^2^ statistic, which measures the percentage of total variation across studies due to heterogeneity, was examined (*I*^2^ > 50% was considered heterogeneity). If significant heterogeneity was observed among the studies, the random-effects model was used; otherwise, the fixed effects model was utilized. Begg's test was employed to assess the publication bias (45). A *p*-value < 0.05 was considered as statistically significant. Moreover, subgroup analysis was employed for both dietary and circulating vitamin E level, respectively.

## Results

### Study Identification and Selection

**Figure 1:**
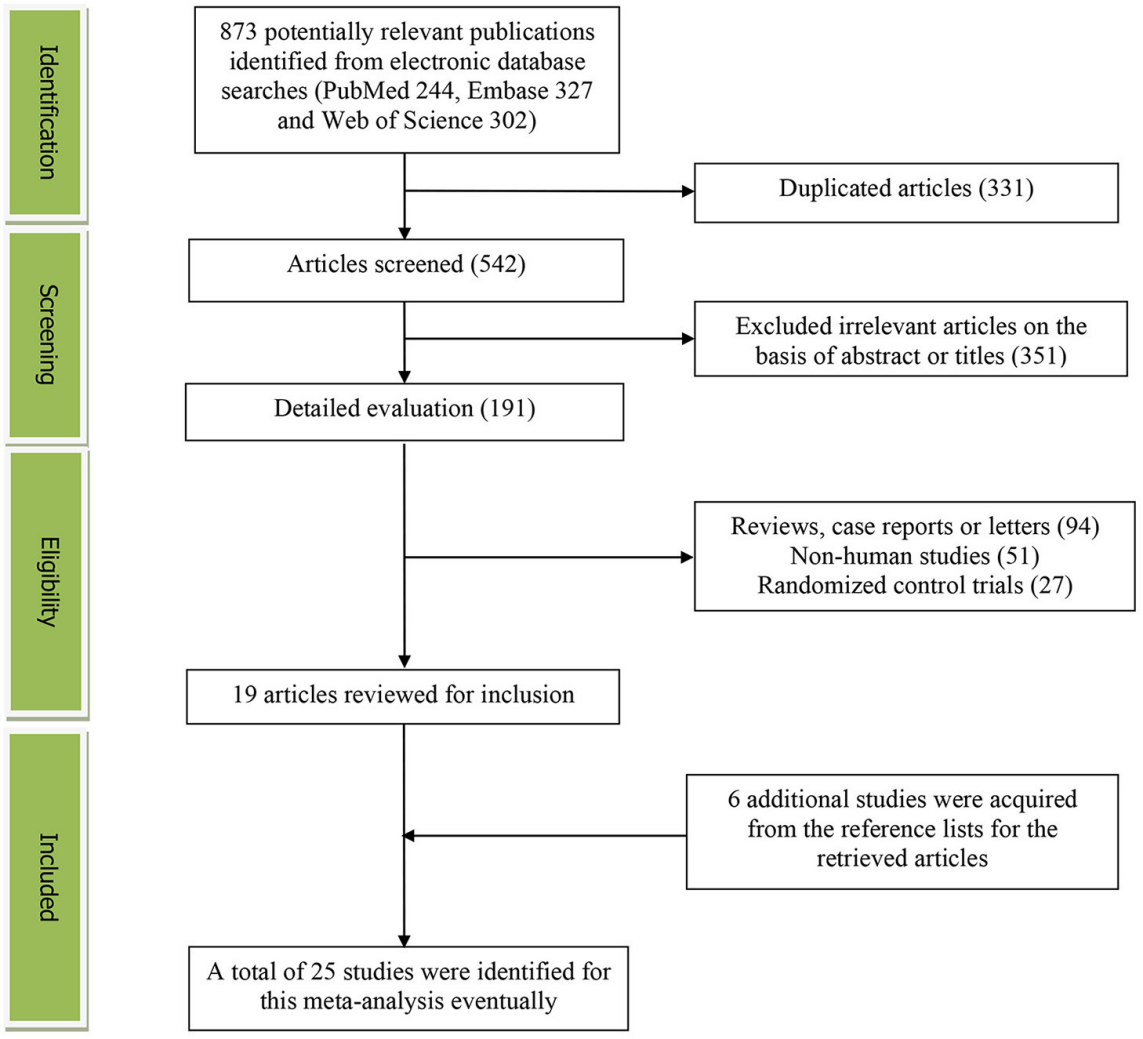
The detailed flow diagram of the study identification and selection in this meta-analysis.

The detailed flow diagram of the study identification and selection was presented in Figure 1. A total of 873 potentially relevant articles (244 for PubMed, 327 for Embase and 302 for Web of Science) were retrieved during the initial literature search. After eliminating 331 duplicated articles, 542 articles were screened. 351 irrelevant studies were excluded according to the titles and abstracts. Then, 94 reviews, case reports or letters, 51 non-human studies, 27 randomized control trials studies were removed. Thereafter, 6 additional studies were acquired from the reference lists for the retrieved articles. Eventually, a total of 25 studies were selected for this meta-analysis (17–41).

### Study Characteristics

Table 1 showed the main characteristics of the included studies. These studies were published between 2003 and 2021. Among which, 14 studies were performed in Asian countries [Korea (18, 19, 21, 35–39), China (29, 30, 33, 40), Iran (31, 41) and Saudi Arabia (28)], and 4 ones were conducted in European countries [Poland (34, 38), France (20) and Finland (24)]. The other 7 studies were from US (17, 23, 25, 26, 32), Brazil (22) and Nigeria (27). Both male and female participants were considered in all included studies, except for Bruscate's and Cho's study (21, 22). The sample size ranged from 20 to 10,351 for a total number of 51,276. The dietary vitamin E level was assessed by food-frequency questionnaire (FFQ) in 5 studies (17, 19, 26, 33, 41), and 24 h or 3-day recall method in 18 studies (18, 20, 22, 23, 25, 27–32, 34–40), and 4-day record in 1 study (24). The circulating vitamin E level was assessed by high performance liquid chromatography (HPLC) in 11 studies (17, 18, 20, 21, 23, 25, 27, 30, 32, 38, 39), and spectrophotometric method in 1 study (34). The criteria for MetS were National Cholesterol Education Program-Adult Treatment Panel III (NCEP ATP III) in 16 (17–21, 23, 24, 27, 29, 30, 35–37, 39–41) and International Diabetes Federation (IDF) in 6 studies (22, 25, 28, 31, 34, 38). The others utilized American Heart Association (AHA) (26, 33) or Joint Interim Statement (JIS) (32), respectively.

**Table 1:** Characteristics of the individual studies included in this meta-analysis.

**Reference**	**Location**	**Age years**	**Gender**	**Sample Size**	**Study design**	**Adjustments**	**Exposure assessment**	**Category of exposure**	**Effect Estimates (RR or SMD)**	**Diagnostic criteria of MetS**	**NOS**
Ford (17)	US	>20	Both	8,808	Cross-sectional	NA	FFQ and HPLC	Control subjects MetS subjects Control subjects MetS subjects	Dietary vitamin E 9.9 (9.5, 10.3) 9.4 (8.8, 10.0) Circulating vitamin E 25.9 (25.5, 26.4) 30.1 (29.2, 30.9)	NCEP ATP III	8
Kim (18)	Korea	>60	Both	404	Cross-sectional	Age, BMI, energy intake, smoking status, alcohol, physical activity, vitamin, and mineral supplements	24 h recall and HPLC	Male Control subjects MetS subjects	Dietary vitamin E 8.6 (7.0, 10.2) 7.0 (5.6, 8.4)	NCEP ATP III	7
								Female Control subjects MetS subjects	Dietary vitamin E 8.6 (7.6, 9.6) 6.7 (5.9, 7.5)		
								Dietary vitamin E Male Quartiles 1 Quartiles 2 Quartiles 3 Quartiles 4	RR 1.0 0.71 (0.25, 2.1) 0.94 (0.33, 2.67) 0.54 (0.18, 1.62)		
								Female Quartiles 1 Quartiles 2 Quartiles 3 Quartiles 4	RR 1.0 1.0 (0.52, 1.93) 0.52 (0.43, 1.58) 0.50 (0.26, 0.98)		
								Control subjects MetS subjects	Circulating vitamin E 11.7 (10.5, 12.9) 10.8 (10.0, 11.6)		
								Circulating vitamin E Male Tertiles 1 Tertiles 2 Tertiles 3	RR 1.0 1.3 (0.2, 7.3) 3.5 (0.7, 17.7)		
								Female Tertiles 1 Tertiles 2 Tertiles 3	RR 1.0 1.1 (0.5, 2.9) 0.8 (0.3, 1.9)		
Kim (19)	Korea	Middle-aged	Both	688	Cross-sectional	NA	FFQ	Male Control subjects MetS subjects	Dietary vitamin E 7.9 (7.5, 8.3) 7.7 (7.3, 8.2)	NCEP ATP III	6
								Female Control subjects MetS subjects	Dietary vitamin E 8.7 (8.1, 9.3) 8.1 (7.6, 8.6)		
Czernichow (20)	France	49	Both	5,520	Cohort	Age, sex, intervention group, educational level, smoking status, physical activity and alcohol consumption	HPLC	Circulating vitamin E Tertiles 1 Tertiles 2 Tertiles 3	RR 1.0 0.94 (0.64, 1.38) 1.02 (0.70, 1.49)	NCEP ATP III	9
Cho (21)	Korea	>20	Male	163	Cross-sectional	NA	HPLC	Control subjects MetS subjects	Circulating vitamin E 1,153.3 (1,100.7, 1,206.0) 1,315.7 (1,148.8, 1,482.5)	NCEP ATP III	6
Bruscate (22)	Brazil	69.3	Female	284	Cross-sectional	Age, smoking, education, physical activity and dietary fiber	24 h recall	Control subjects MetS subjects	Dietary vitamin E 17.8 (16.7, 18.9) 17.4 (15.7, 19.1)	IDF	7
								Dietary vitamin E Quartiles 1 Quartiles 2 Quartiles 3 Quartiles 4	RR 1.0 0.52 (0.25, 1.08) 0.65 (0.32, 1.33) 0.70 (0.34, 1.42)		
Beydoun (23)	US	20–85	Both	3,202	Cross-sectional	Age, sex, race/ethnicity, marital status, educational level, PIR, smoking status, total energy intake, alcohol, caffeine, b-carotene, vitamin C, vitamin E, and dietary supplement use, serum levels of folate, tHcy, vitamin B12, 25(OH)D, total cholesterol, and TG	24 h recall and HPLC	Male Control subjects MetS subjects	Dietary vitamin E 8.2 (7.8, 8.6) 8.0 (7.6, 8.4)	NCEP ATP III	8
								Female Control subjects MetS subjects	Dietary vitamin E 6.6 (6.4, 6.8) 6.1 (5.7, 6.5)		
								Male Control subjects MetS subjects	Circulating vitamin E 28.5 (27.5, 29.5) 33.8 (32.0, 35.6)		
								Female Control subjects MetS subjects	Circulating vitamin E 28.2 (27.2, 29.2) 35.3 (33.5, 37.1)		
								Circulating vitamin E Quartiles 1 Quartiles 2 Quartiles 3 Quartiles 4	RR 1.0 0.79 (0.41, 1.52) 1.54 (0.84, 2.84) 1.09 (0.44, 2.67)		
Kouki (24)	Finland	57–78	Both	1,334	Cross-sectional	Age, alcohol consumption, smoking, education and VO_2_ max	4-day food record	Male Control subjects MetS subjects	Dietary vitamin E 11.1 (10.6, 11.6) 9.9 (9.0, 10.8)	NCEP ATP III	8
								Female Control subjects MetS subjects	Dietary vitamin E 9.8 (9.2, 10.4) 9.5 (8.6, 10.4)		
								Dietary vitamin E Male <10 mg/d >10 mg/d	RR 1.0 0.99 (0.96, 1.03)		
								Female <10 mg/d >10 mg/d	RR 1.0 1.01 (0.98, 1.05)		
Beydoun (25)	US	12–19	Both	1,339	Cross-sectional	NA	24 h recall and HPLC	Control subjects MetS subjects	Dietary vitamin E 6.4 (6.2, 6.6) 5.9 (4.3, 7.5)	IDF	7
								Control subjects MetS subjects	Circulating vitamin E 18.6 (18.2, 19.0) 20.2 (18.0, 22.4)		
de Oliveira Otto (26)	US	45–84	Both	3,828	Cohort	Energy intake, age, sex, race-ethnicity, education, study center, alcohol intake, physical activity, BMI, fiber intake, cigarette smoking, dietary supplement use, the ratio of polyunsaturated fat intake and saturated fat intake, Mg, Zn, heme iron, non-heme iron, and antioxidant intake	FFQ	Dietary vitamin E Quintiles 1 Quintiles 2 Quintiles 3 Quintiles 4 Quintiles 5	RR 1.0 1.00 (0.79, 1.26) 1.03 (0.81, 1.31) 0.84 (0.65, 1.09) 0.76 (0.56, 1.03)	AHA	8
Odum (27)	Nigeria	50	Both	192	Case-control	NA	HPLC	Control subjects MetS subjects	Circulating vitamin E 30.8 (29.6, 32.0) 16.9 (15.9, 17.9)	NCEP ATP III	7
Al-Daghri (28)	Saudi Arabia	19–60	Both	185	Cross-sectional	Age, BMI and physical activity	24 h recall	Control subjects MetS subjects	Dietary vitamin E 2.3 (2.1, 2.5) 2.0 (1.8, 2.2)	IDF	7
								Dietary vitamin E Quartiles 1 Quartiles 2 Quartiles 3 Quartiles 4	RR 1.0 0.20 (0.07, 0.60) 0.14 (0.05, 0.40) 0.17 (0.06, 0.51)		
Motamed (31)	Iran	35–65	Both	3,800	Cross-sectional	Sex, age, physical activity level, smoking, past medical history, energy intake, and BMI	24 h recall	Male Control subjects MetS subjects	Dietary vitamin E 14.8 (14.2, 15.4) 16.6 (15.9, 17.3)	IDF	7
								Female Control subjects MetS subjects	Dietary vitamin E 14.7 (14.3, 15.1) 13.8 (13.4, 14.2)		
								Dietary vitamin E Quintiles 1 Quintiles 2 Quintiles 3 Quintiles 4 Quintiles 5	RR 1.0 0.96 (0.70, 1.10) 0.86 (0.60, 1.00) 1.06 (0.80, 1.30) 0.90 (0.70, 1.10)		
Bian (29)	China	30–70	Both	258	Cross-sectional	NA	24 h recall	Control subjects MetS subjects	Dietary vitamin E 29.9 (28.4, 31.4) 27.5 (25.9, 29.1)	NCEP ATP III	8
Li (30)	China	18–65	Both	550	Cross-sectional	Age and sex	3-day food record and HPLC	Control subjects MetS subjects	Dietary vitamin E 45.7 (43.2, 48.2) 45.1 (42.1, 48.1)	NCEP ATP III	8
								Dietary vitamin E Quartiles 1 Quartiles 2 Quartiles 3 Quartiles 4 Control subjects	RR 1.0 0.65 (0.37,1.04) 0.85 (0.51–1.41) 0.62 (0.37–1.04) Circulating vitamin		
								MetS subjects	E 12.1 (11.7, 12.5) 12.9 (12.2, 13.6)		
								Circulating vitamin E Quartiles 1 Quartiles 2 Quartiles 3 Quartiles 4	RR 1.0 0.44 (0.15,1.25) 0.88 (0.32–2.44) 1.58 (0.55–4.54)		
Mah (32)	US	24–40	Both	20	Case-control	NA	HPLC	Control subjects MetS subjects	Circulating vitamin E 22.2 (19.5, 24.9) 23.9 (21.9, 25.9)	JIS	5
Wei (33)	China	18–84	Both	2,069	Cross-sectional	Age, sex, cigarette smoking, alcohol, drinking, nutritional supplementary, activity level, dietary energy intake, fiber intake and protein intake	FFQ	Control subjects MetS subjects	Dietary vitamin E 29.8 (29.1, 30.6) 30.7 (28.8, 32.6)	AHA	7
								Dietary vitamin E Quartiles 1 Quartiles 2 Quartiles 3 Quartiles 4	RR 1.0 1.07 (0.77, 1.50) 0.98 (0.67, 1.41) 1.20 (0.77, 1.87)		
Godala (34)	Poland	30–65	Both	273	Case-control	NA	3-day food record and spectrophotometric method	Control subjects MetS subjects	Dietary vitamin E 9.33 (8.27, 10.39) 8.85 (8.03, 9.67)	IDF	7
								Control subjects MetS subjects	Circulating vitamin E 25.49 (24.86, 26.12) 12.47 (12.08, 12.86)		
Lim (36)	Korea	Middle-aged	Both	143	Cross-sectional	Not mentioned	3-day food record	Control subjects MetS subjects	Dietary vitamin E 12.6 (11.5, 13.7) 12.7 (11.3, 14.1)	NCEP ATP III	6
Ahn (35)	Korea	30–60	Both	614	Cross-sectional	Age, smoking, alcohol consumption and physical activity	3-day food record	Male Control subjects MetS subjects	Dietary vitamin E 3.5 (3.3, 3.7) 3.3 (3.2, 3.4)	NCEP ATP III	7
								Female Control subjects MetS subjects	Dietary vitamin E 3.5 (3.3, 3.7) 3.8 (3.5, 4.1)		
								‘Dietary vitamin E Male Tertile 1 Tertile 2 Tertile 3	RR 1.0 1.00 (0.56, 1.76) 0.52 (0.30, 0.92)		
								Female Tertile 1 Tertile 2 Tertile 3	RR 1.0 1.16 (0.65, 2.09) 1.70 (0.94, 3.08)		
Ahn (37)	Korea	19–64	Both	10,351	Cross-sectional	Age, BMI, alcohol consumption, smoking, physical activity, household income, education level and energy intake	24 h recall	Dietary vitamin E Male Tertile 1 Tertile 2 Tertile 3	RR 1.0 0.88 (0.71, 1.11) 0.76 (0.60, 0.96)	NCEP ATP III	8
								Female Tertile 1 Tertile 2 Tertile 3	RR 1.0 0.87 (0.67, 1.14) 1.02 (0.79, 1.31)		
Godala (38)	Poland	57	Both	332	Cross-sectional	NA	24 h recall and HPLC	Control subjects MetS subjects	Dietary vitamin E 9.31 (8.26, 10.35) 9.01 (8.37, 9.66)	IDF	7
								Control subjects MetS subjects	Circulating vitamin E 23.67 (22.88, 24.46) 14.22 (13.93, 14.51)		
Kim (39)	Korea	47.1	Both	5,885	Cross-sectional	Age, sex, residence, household income, education, alcohol consumption, smoking status, physical activity, hs-CRP and BMI	HPLC	Circulating vitamin E Quartiles 1 Quartiles 2 Quartiles 3 Quartiles 4	RR 1.0 1.30 (0.97, 1.74) 1.71 (1.30, 2.25) 2.56 (1.95, 3.35)	NCEP ATP III	9
Peng (40)	China	>99	Both	992	Cohort	Aex, marital status, physical activity, smoking status, alcohol intake, family history of chronic diseases and daily total energy intake	24 h recall	Control subjects MetS subjects	Dietary vitamin E 17.74 (10.45, 25.03) 12.18 (9.00, 15.36)	NCEP ATP III	7
Zaeemzadeh (41)	Iran	30	Both	42	Case-control	NA	FFQ	Control subjects MetS subjects	Dietary vitamin E 17.21 (14.49, 19.93) 6.17 (2.01, 10.33)	NCEP ATP III	6

### RR of MetS for the Highest vs. Lowest Dietary Vitamin E Level

**Figure 2:**
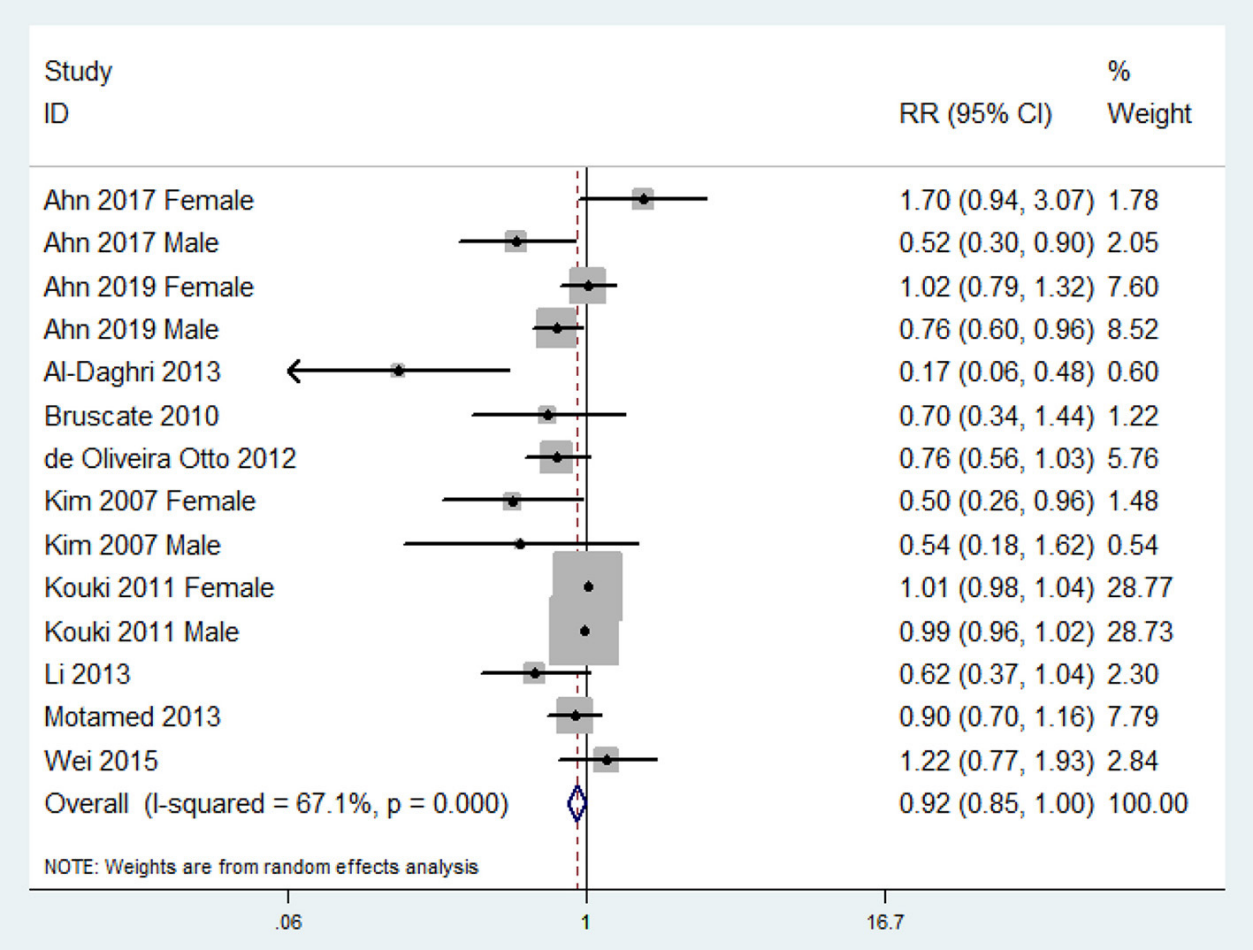
Forest plot of meta-analysis: Overall multi-variable adjusted RR of MetS for the highest vs. lowest category of dietary vitamin E level.

The overall multi-variable adjusted RR demonstrated that the dietary vitamin E level was negatively associated with MetS (RR = 0.92, 95%CI: 0.85–1.00; *P* = 0.044) (Figure 2). A substantial level of heterogeneity among various studies was obtained (*P* < 0.001, I^2^ = 67.1%). The Begg's rank-correlation test showed no evidence of publication bias (*P* = 0.189). The results of subgroup analysis were presented in Table 2. The aforementioned results only existed in studies with the adjustment of BMI (RR = 0.75, 95%CI: 0.59 to 0.94; *P* = 0.01), physical activity (RR = 0.76, 95%CI: 0.61–0.95; *P* = 0.02), and energy intake (RR = 0.86, 95%CI: 0.76–0.97; *P* = 0.01), respectively.

**Table 2:** Subgroup analysis of MetS for the highest vs. lowest dietary vitamin E level category.

**Stratification**	**Number of studies**	**Pooled RR**	**95% CI**	**P value**	**Heterogeneity**
All studies	10	0.92	0.85, 1.00	*P* = 0.04	*P* < 0.001; *I*^2^ = 67%
**Adjustment of BMI**					
Adjusted	5	0.75	0.59, 0.94	*P* = 0.01	*P* = 0.02; *I*^2^ = 61%
Unadjusted	5	0.99	0.93, 1.06	*P* = 0.79	*P* = 0.03; *I*^2^ = 58%
**Adjustment of physical activity**					
Adjusted	7	0.76	0.61, 0.95	*P* = 0.02	*P* = 0.004; *I*^2^ = 63%
Unadjusted	3	1	0.98, 1.02	*P* = 0.98	*P* = 0.18; *I*^2^ = 38%
**Adjustment of energy intake**					
Adjusted	5	0.86	0.76, 0.97	P = 0.01	P = 0.17; I^2^ = 34%
Unadjusted	5	0.96	0.88, 1.05	*P* = 0.42	*P* < 0.001; *I*^2^ = 76%
**Adjustment for vitamin E supplement**					
Adjusted	3	0.8	0.64, 1.01	*P* = 0.06	*P* = 0.12; *I*^2^ = 48%
Unadjusted	7	0.94	0.87, 1.02	*P* = 0.14	*P* < 0.001; *I*^2^ = 70%
**Study design**					
Cross-sectional	9	0.93	0.86, 1.01	*P* = 0.10	*P* < 0.001; *I*^2^ = 67%
Cohort	1	0.76	0.56, 1.03	/	/

### SMD of Dietary Vitamin E Level for MetS vs. Control Subjects

**Figure 3:**
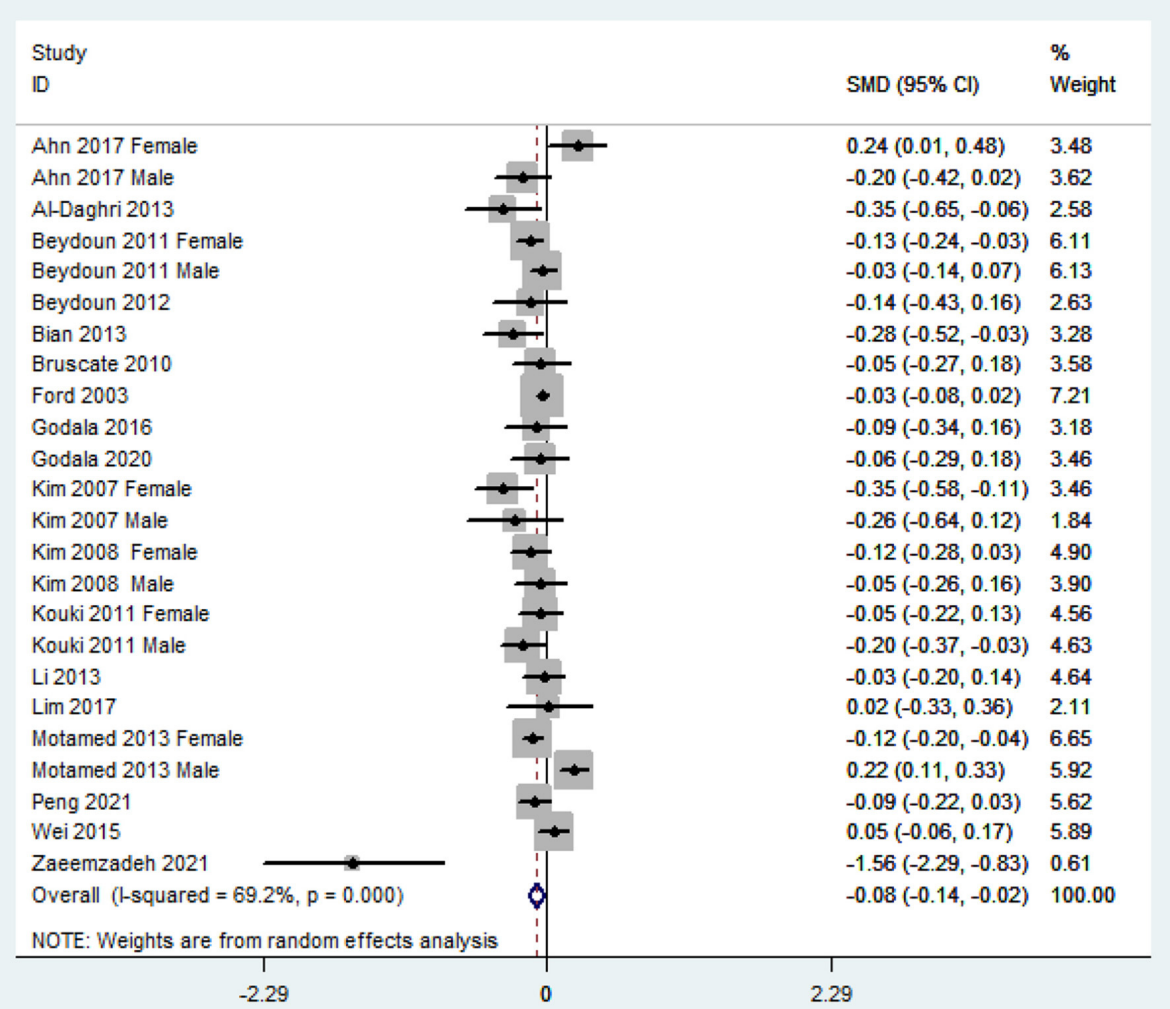
Forest plot of meta-analysis: SMD of dietary vitamin E level for MetS vs. control subjects.

The overall combined SMD showed that the dietary vitamin E level in MetS was lower than that in control subjects (SMD = −0.08, 95%CI: −0.14 to −0.02; *P* = 0.006) (Figure 3). A substantial level of heterogeneity was obtained among the various studies (*P* < 0.001, *I*^2^ = 69.2%). The Begg's rank-correlation test showed no evidence of publication bias (*P* = 0.07). The results of subgroup analysis were presented in Table 3. The aforementioned results only existed in female (SMD = −0.10, 95%CI: −0.20 to 0.00; *P* = 0.05), NCEP ATP III (SMD = −0.11, 95%CI: −0.07 to −0.04; *P* = 0.002), 24 h or 3 days recall (SMD = −0.09, 95%CI: −0.16 to −0.02; *P* = 0.01) and high-quality (SMD = −0.07, 95%CI: −0.13 to −0.01; *P* = 0.02) studies, respectively.

**Table 3:** Subgroup analysis for SMD of dietary vitamin E level in MetS vs. control subjects.

**Stratification**	**Number of studies**	**Pooled SMD**	**95% CI**	***P*-value**	**Heterogeneity**
All studies	18	−0.08	−0.14, −0.02	*P* = 0.006	*P* < 0.001; *I*^2^ = 69%
**Gender**					
Male	6	−0.06	−0.22, 0.09	*P* = 0.44	*P* < 0.001; *I*^2^ = 80%
Female	6	−0.10	−0.20, 0.00	*P* = 0.05	*P* = 0.02; *I*^2^ = 63%
**Diagnostic criteria of MetS**					
NCEP ATP III	11	−0.11	−0.07, −0.04	*P* = 0.002	*P* < 0.001; *I*^2^ = 63%
Other	7	−0.04	−0.16, 0.08	*P* = 0.51	*P* < 0.001; *I*^2^ = 77%
**Geographical region**					
Asia	11	−0.10	−0.21, 0.00	*P* = 0.05	*P* < 0.001; *I*^2^ = 79%
Non-Asia	7	−0.06	−0.09, −0.02	*P* = 0.002	*P* = 0.62; *I*^2^ = 0%
**Exposure assessment**					
FFQ	4	−0.09	−0.23, 0.06	*P* = 0.24	*P* = 0.001; *I*^2^ = 80%
24 h or 3 days recall	14	−0.09	−0.16, −0.02	*P* = 0.01	*P* < 0.001; *I*^2^ = 66%
**Study quality**					
High-quality	15	−0.07	−0.13, −0.01	*P* = 0.02	*P* < 0.001; *I*^2^ = 67%
Low-quality	3	−0.24	−0.56, 0.08	*P* = 0.14	*P* = 0.001; *I*^2^ = 81%

### RR of MetS for the Highest vs. Lowest Circulating Vitamin E Level

**Figure 4:**
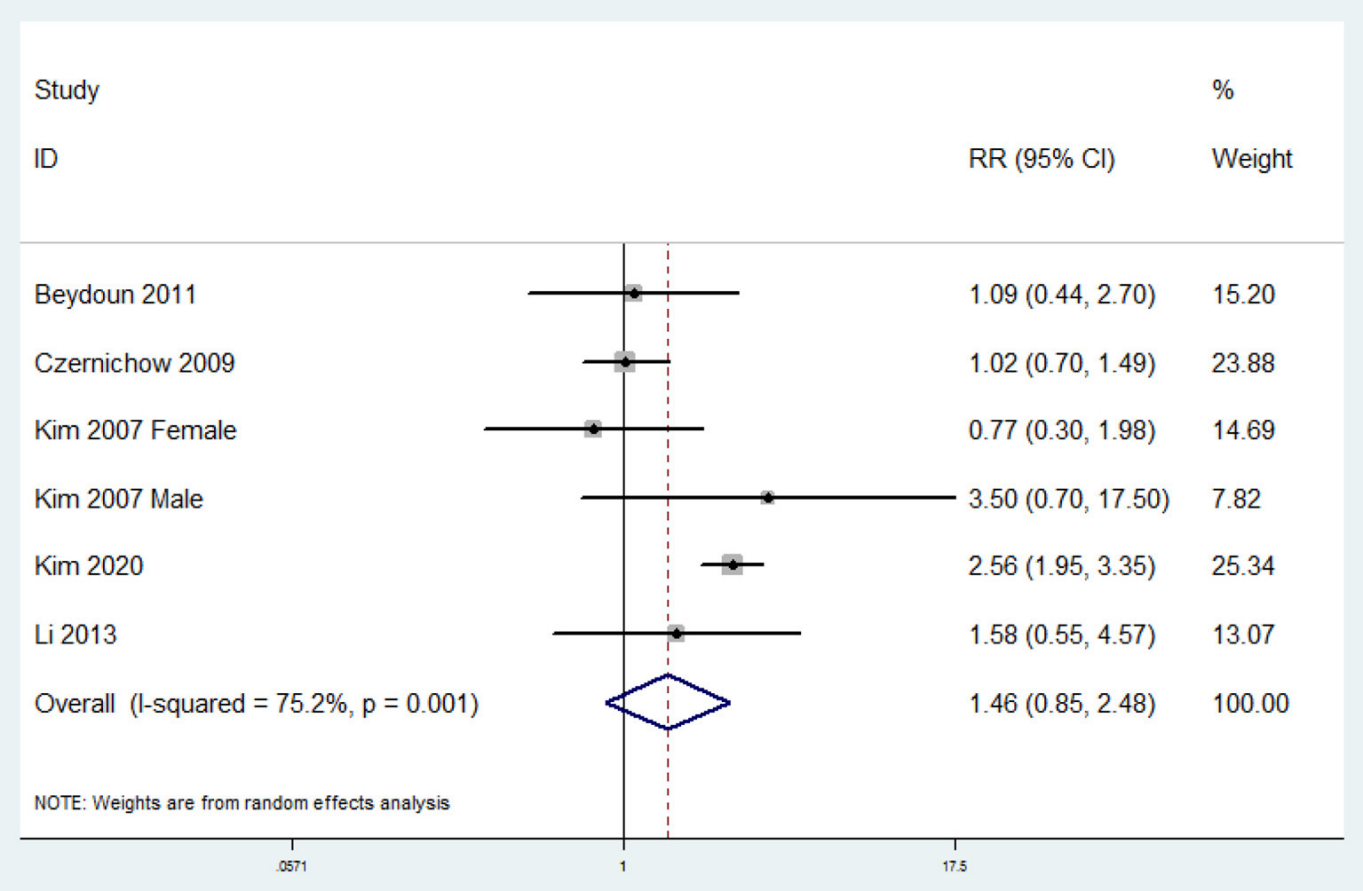
Forest plot of meta-analysis: Overall multi-variable adjusted RR of MetS for the highest vs. lowest category of circulating vitamin E level.

The overall multi-variable adjusted RR showed no significant relationship between circulating vitamin E level and MetS (RR = 1.46, 95%CI: 0.85–2.48; *P* = 0.168) (Figure 4). A substantial level of heterogeneity was obtained among various studies (*P* = 0.001, *I*^2^ = 75.2%). The Begg's rank-correlation test showed no evidence of publication bias (*P* = 0.707). The results of subgroup analysis were presented in Table 4.

**Table 4:** Subgroup analysis of MetS for the highest vs. lowest circulating vitamin E level category.

**Stratification**	**Number of studies**	**Pooled RR**	**95% CI**	**P value**	**Heterogeneity**
All studies	5	1.46	0.85, 2.48	*P* = 0.17	*P* = 0.001; *I*^2^ = 75%
**Adjustment of BMI**					
Adjusted	2	1.84	0.78, 4.38	*P* = 0.16	*P* = 0.05; *I*^2^ = 67%
Unadjusted	3	1.07	0.77, 1.49	*P* = 0.67	*P* = 0.75; *I*^2^ = 0%
**Adjustment of physical activity**					
Adjusted	2	1.84	0.78, 4.38	*P* = 0.16	*P* = 0.05; *I*^2^ = 67%
Unadjusted	3	1.07	0.77, 1.49	*P* = 0.67	*P* = 0.75; *I*^2^ = 0%
**Adjustment of energy intake**					
Adjusted	2	1.11	0.61, 2.04	*P* = 0.73	*P* = 0.28; *I*^2^ = 21%
Unadjusted	3	1.62	0.79, 3.35	*P* = 0.19	P < 0.001; *I*^2^ = 87%
**Adjustment for vitamin E supplement**					
Adjusted	2	1.11	0.61, 2.04	*P* = 0.73	*P* = 0.28; *I*^2^ = 21%
Unadjusted	3	1.62	0.79, 3.35	*P* = 0.19	P < 0.001; *I*^2^ = 87%
Study design					
Cross-sectional	4	1.65	0.94, 2.89	*P* = 0.08	*P* = 0.06; *I*^2^ = 55%
Cohort	1	1.02	0.70, 1.49	/	/

### SMD of Circulating Vitamin E Level for MetS vs. Control Subjects

**Figure 5:**
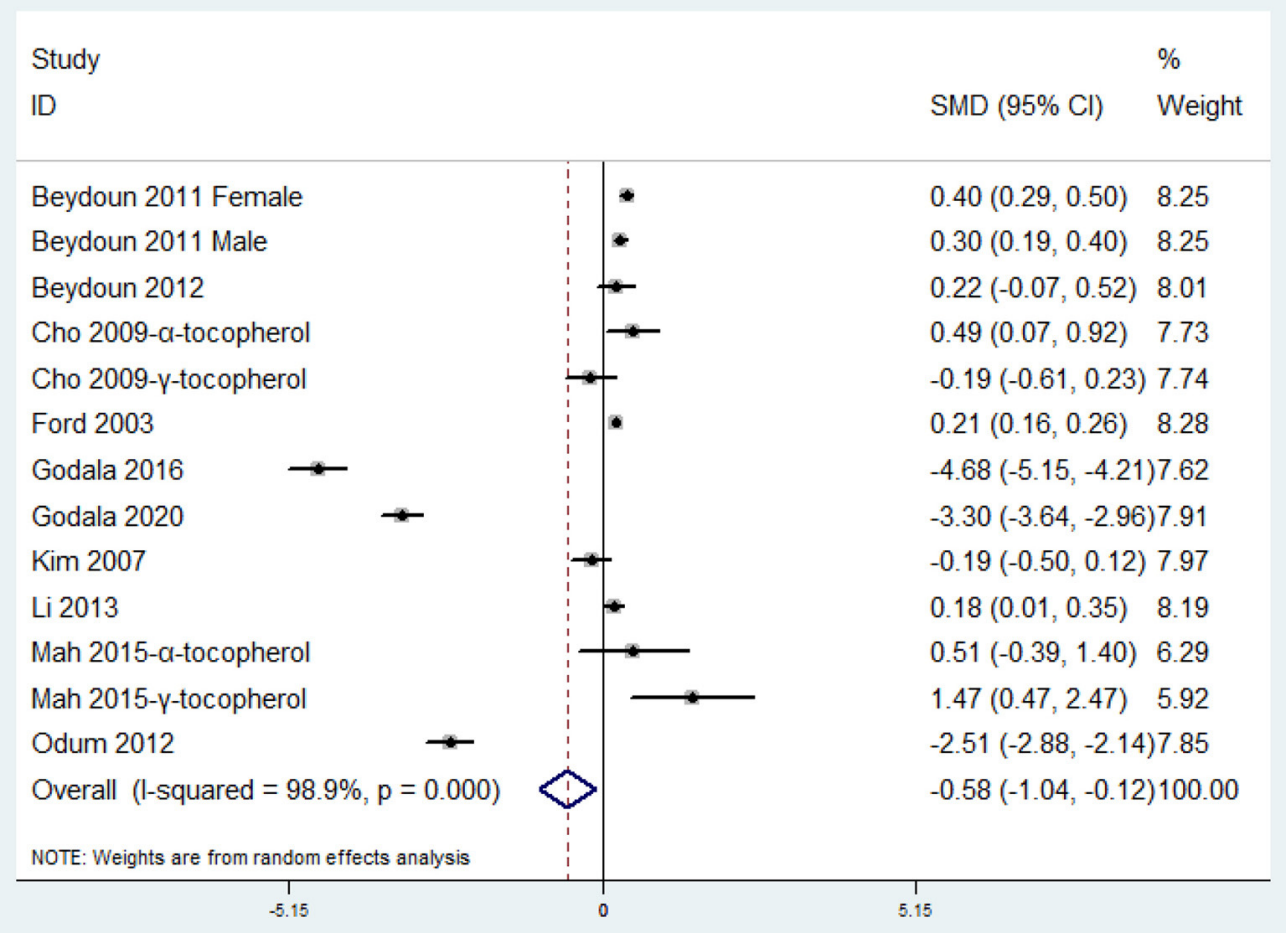
Forest plot of meta-analysis: SMD of circulating vitamin E level for MetS vs. control subjects.

The overall combined SMD showed that the circulating vitamin E level in MetS was lower than that in control subjects (SMD = −0.58, 95%CI: −1.04 to −0.13; *P* = 0.013) (Figure 5). A substantial level of heterogeneity was obtained among the various studies (*P* < 0.001, *I*^2^ = 98.9%). The Begg's rank-correlation test showed no evidence of publication bias (*P* = 0.246). The results of subgroup analysis were presented in Table 5. The aforementioned results only existed in spectrophotometric method (SMD = −4.67, 95%CI: −5.14 to −4.20) and high-quality study (SMD = −1.00, 95%CI: −1.54 to −0.45; *P* < 0.001), but lost in α-tocopherol (SMD = 0.08, 95%CI: −0.16 to 0.33; *P* = 0.50) and γ-tocopherol (SMD = 0.54, 95%CI: −1.03 to 2.11; *P* = 0.50), respectively.

**Table 5:** Subgroup analysis for SMD of circulating vitamin E level in MetS vs. control subjects.

**Stratification**	**Number of studies**	**Pooled SMD**	**95% CI**	**P-value**	**Heterogeneity**
All studies	10	−0.58	−1.04, −0.13	*P* = 0.01	*P* < 0.001; *I*^2^ = 99%
**Assessment of outcome**					
HPLC	9	−0.24	−0.64, 0.12	*P* = 0.17	*P* < 0.001; *I*^2^ = 99%
Spectrophotometric method	1	−4.67	−5.14, −4.20	/	/
**Study quality**					
High quality	8	−1.00	−1.54, −0.45	*P* < 0.001	*P* < 0.001; *I*^2^ = 99%
Low quality	2	0.44	−0.15, 1.02	*P* = 0.14	*P* = 0.01; *I*^2^ = 73%
**Type of tocopherol**					
α-tocopherol	3	0.08	−0.16, 0.33	*P* = 0.50	*P* = 0.03; *I*^2^ = 72%
γ-tocopherol	2	0.54	−1.03, 2.11	*P* = 0.50	*P* = 0.004; *I*^2^ = 88%

## Discussion

In the present meta-analysis, a total of 25 observational studies were identified for examination. The pooled analysis showed that the dietary vitamin E level was inversely associated with MetS.

Since both the oxidative stress and inflammation plays important role in the pathophysiology of MetS (46), the negative relationship between dietary vitamin E level and MetS may be mainly attributed to the antioxidant and anti-inflammatory property of vitamin E (12, 47, 48). Consistently, the clinical trial evidence demonstrates that the vitamin E supplementation may ameliorate MetS via anti-inflammation and anti-oxidative stress (49, 50). Moreover, some experimental animal studies have also indicated the beneficial impact of vitamin E intake on the MetS-related context (13–16). Nevertheless, very limited prospective cohort studies are identified for meta-analysis. The dietary vitamin E level is measured after MetS event occurs in cross-sectional/case-control studies. The factors that matter the dietary vitamin E level may change after MetS, which may reverse the causality. In addition, the significant heterogeneity has also been specified (*I*^2^ = 67.1%). Therefore, these above issues should be addressed by further well-designed prospective cohort studies.

In contrast to dietary vitamin E (a general/overall estimation that calculated from the FFQ or recall method), the issue of circulating vitamin E level is rather complicated. On one hand, vitamin E is composed of 8 similar structure compounds: 4 tocopherol and 4 tocotrienol derivatives including α-, β-, γ-, δ-tocopherol and tocotrienol, respectively. All vitamin E isomers from dietary or supplementary sources are absorbed and delivered to the liver. Only α-tocopherol is preferentially recognized by the α-tocopherol transfer protein for incorporation into circulating plasma, whereas the other tocopherol and tocotrienol isomers are mostly excreted (51, 52). Indeed, the concentration of tocotrienol is significantly lower than that of α-tocopherol (52). This is the main reason why α-tocopherol is currently severed as the standard to estimate human vitamin E requirements (53). However, it is increasingly acknowledged that tocopherol and tocotrienol serve different biological functions (tocotrienol may result in superior therapeutic properties than tocopherol), which has greatly challenged the accuracy of α-tocopherol estimation alone (54–56). Unfortunately, only several studies reported the exposure as “tocopherol” (21, 32, 39) (most studies reported the exposure as “circulating vitamin E” directly). The combination of tocopherol/tocotrienol may probably preclude a clear clarification for this issue. On the other hand, chronic inflammation and oxidative stress caused by MetS may lower the circulating antioxidant and anti-inflammatory agent level (57). In turn, the circulating antioxidant and anti-inflammatory agent level may also increase to cope with chronic inflammation and oxidative stress caused by MetS (58). As a consequence, the level of circulating vitamin E might be dynamic in MetS condition. Taken together, the specification of tocopherol/tocotrienol and potential feedback system should be considered for circulating vitamin E in further studies.

The epidemiological data has demonstrated that BMI, physical activity and energy intake is associated with both dietary vitamin E and MetS (59–63). Indeed, our finding is lost in studies without adjustment of these factors (Table 2). The adjustment of confounding factors can exclude the effect on both exposure and outcome (leave the direct relationship between vitamin E and MetS). Therefore, BMI, physical activity and energy intake may be adjusted when further study is employed. In addition, the lower dietary vitamin E level in MetS is only obtained in females, NCEP ATP III, 24 h or 3 days recall and high-quality studies (Table 3). Although females are more precise and reliable in completing the exposure assessment, some genetic gender differences with the diet-related pathology of MetS cannot be fully excluded (64). Moreover, the diagnostic criteria of MetS and exposure assessment may also influence the results. Since a substantial improvement may exist in laboratorial techniques to assess the circulating vitamin E level, a subgroup analysis for assessment method has also been employed. Interestingly, the results only exist in spectrophotometric method, but not HPLC. Notably, the spectrophotometric method is utilized in only one study (34). Taken together, more study with appropriate confounding factors adjustment and different circulating vitamin E assessment is needed.

The strengths of the present meta-analysis are mainly reflected in the following aspects: First, this is the first meta-analysis of observational studies on the associations of dietary and circulating vitamin E level with MetS. Second, our findings for dietary vitamin E level and MetS are consistence with current corresponding experimental and clinical studies. The limitations should also be acknowledged. First, the substantial level of heterogeneity might have distorted the reliability of our results (especially for circulating vitamin E level). Second, due to the limitation in the relevant literature, very few prospective cohort studies are identified totally (precluded causal relationships). Third, the classification of exposure vary greatly among individuals. For example, “tertiles,” “quartiles” and “quintiles” are employed to classify the dietary/circulating vitamin E level. Fourth, the selection of adjusted factors and definition of MetS are not uniform. Last but not the least, very few studies have considered the isomers of tocopherol/tocotrienol. These limitations may weaken the significance of this study.

## Conclusions

The results of this meta-analysis suggest that the dietary vitamin E level may be inversely associated with MetS. On the other hand, current evidence is still insufficient to conclude a relationship between the circulating vitamin E level and MetS. More well-designed prospective cohort studies with the specification of circulating tocopherol/tocotrienol are needed to address the issues further.

## Data Availability Statement

The raw data supporting the conclusions of this article will be made available by the authors, without undue reservation.

## Author Contributions

YZ, DZ, and JL conceived the idea and drafted this meta-analysis. ZL and QL performed the statistical analysis. YL and JD selected and retrieved relevant papers. HG assessed each study. YZ, DZ, and JL was the guarantor of the overall content. All authors revised and approved the final manuscript.

## Funding

This study was supported by National Natural Science Foundation of China (82102581), National Postdoctoral Science Foundation of China (2021M693562), Provincial Outstanding Postdoctoral Innovative Talents Program of Hunan (2021RC2020), Provincial Natural Science Foundation of Hunan (2019JJ40517), Young Investigator Grant of Xiangya Hospital, Central South University (2020Q14), and FuQing Postdoc Program of Xiangya Hospital, Central South University (176).

## Conflict of Interest

The authors declare that the research was conducted in the absence of any commercial or financial relationships that could be construed as a potential conflict of interest.

## Publisher's Note

All claims expressed in this article are solely those of the authors and do not necessarily represent those of their affiliated organizations, or those of the publisher, the editors and the reviewers. Any product that may be evaluated in this article, or claim that may be made by its manufacturer, is not guaranteed or endorsed by the publisher.
